# Perspectives in HPV Secondary Screening and Personalized Therapy Basing on Our Understanding of HPV-Related Carcinogenesis Pathways

**DOI:** 10.1155/2020/2607594

**Published:** 2020-03-25

**Authors:** Aleksander Celewicz, Marta Celewicz, Michał Michalczyk, Rafał Rzepka

**Affiliations:** ^1^Department of Gynecology and Obstetrics, Collegium Medicum University of Zielona Góra, Poland; ^2^Department of Obstetrics and Gynecology, Pomeranian Medical University, Poland

## Abstract

As cervical cancer is one of the most common malignancies in women worldwide even with present screening methods, the incidence in most developed countries is not decreasing for the last 15-20 years. A shift has been observed in the age of diagnosis in favour of younger women, and treatment of already developed cervical cancer is a challenge for surgeons. It is imperative to find new diagnostic methods for accurately pointing out patients at high risk of developing malignant disease and developing personalized treatment. Since cervical cancer is almost exclusively associated with HPV infection, understanding changes happening in an infected cell may prove invaluable for search of such methods, but it may also prove helpful in the diagnosis and treatment of other anogenital and nasopharyngeal region cancers. This review follows HPV-related changes in infected cell biology to point what potential markers and targets for therapy are in option when dealing with HPV-related diseases.

## 1. Introduction

Cervical cancer is the 5th most common malignancy in women, with over 560 000 cases annually being diagnosed [1]. The incidence of cervical cancer has dropped drastically with introduction of routine screening methods such as the Pap smear. Another breakthrough was the discovery of human papilloma virus (HPV) role in cervical neoplasia [2]. HPV is a member of the *Papillomaviridae* (PV) family, which includes over 200 genotypes specific to humans [3, 4]. They are small circular double-stranded DNA (dsDNA) viruses, encapsulated by capsid. HPV consist of 8 early (E) genes and 2 late (L) genes, with the latter forming the capsid. The gene that is common for the whole family is *L1*. Taxonomy of PV is based on the nucleotide sequence of *L1*. Different genotypes of HPV present different affinities to either squamous epithelium or mucosal membranes, where most commonly they induce low-grade squamous intraepithelial lesions (LSIL), which regress within 2 years in 90% of cases [5].

In some cases, progression to high-grade squamous intraepithelial lesions (HSIL), which are treated as a direct precancerous state, may occur. In this case, surgical intervention is necessary, as potential regression of these lesions is rare, as presented in Table 1.

### 1.1. Papillomaviridae: A Family Older Than *Homo Sapiens*

Taxonomy of Papillomaviridae consists of 5 genera: Alpha, Beta, Gamma, Mu, and Nu. All of them have different tropisms (mucosal or cutaneous). Most of the types from the Gamma genre have no oncogenic potential. This is associated with the ability of the virus to complete its life cycle within the maturing epithelial cells and release new virions to infect another host, without causing any symptoms like condylomas or intraepithelial neoplasia despite being present in mucosal or epithelial cells [6–8]. This is especially vital for cutaneotrophic HPV types, as they are not transmitted as easily as mucosatrophic HPV types, with a strong time/exposure relation [9]. This permission for viral replication in immunocompetent hosts points to a virus-host balance that is a result of immune tolerance, as well as an ability of HPV to evade immune detection [10, 11]. They may be seen as genital warts or nasopharyngeal condylomas. Genital warts, usually caused by low-risk Alpha genre viruses of which most common are types 6 and 11, are associated with high persistence rates when left untreated and a recurrence rate of about 20%. Cure rates also vary depending on therapy (podophyllotoxin, imiquimod, or surgery) between 40 and 80% [12, 13]. Additionally, those types may cause condylomas or recurrent respiratory papillomatosis (RRP) in newborns and juveniles. This recurrent and potentially rapidly progressive and fatal disease [14] may be acquired by the child during labor or through vertical transmission, as DNA HPV was detected in amniotic fluid of individuals with HPV-associated cervical lesions [15]. A variety of papillomaviruses present different strategies for transmission and propagation in the epithelial cells and could also present different types of modulation of host immune system, all of which is an effect of evolutionary adaption [15, 16]. Depending on HPV genotype, the following lesions may be observed in the host during infection as presented in Table 2 [17].

## 2. Role of HPV Infection in Carcinogenesis and Molecular Changes Observed in the Process

As HPV infection is the necessary but insufficient factor in cervical carcinogenesis, several other factors are at play in this oncogenic process [18]: other sexually transmitted infections (Ch. trachomatis and herpes simplex virus), patient immune status, stimulation by exogenous estrogens and gestagenes, and nicotine abuse all add up to the final development of cancer by altering immune response or disrupting cell cycle. Within the plethora of molecular changes in precancerous and cancerous cells, the following article will point to those that are promising in future early diagnosis of precancerous lesions at high risk of progression and potentially useful in patient-tailored therapy.

In the HPV genome, the following genes are distinguished (for detailed information see Table 3):

- Early genes found in the E region
- Regulatory genes *E1* and *E2* that modulate and initiate viral replication
- Oncogenic genes *E5*, *E6*, and *E7* that play an important role in cancerous transformation of infected cells
- Late genes found in the L region, which code structural proteins forming viral capsid: *L1* and *L2*
- Noncoding fragment that performs a control function (long control region (LCR)) [18–27]

Table 3 shows detailed information about HPV proteins and its role in viral life cycle.

HPV infection precedes cancer, and the most common onset point of oncogenic process is development of persistent infection. Depending on the genotype, about 30% of cervical cancers caused by HPV 16 may occur in cells containing an unintegrated (episomal) viral DNA [23, 28, 29] but for other genotypes, it is necessary to integrate the viral genome into the host cell [23, 30, 31]. In the outcome of integration of HPV DNA, a reading frame becomes disrupted in the region of the *E1* or *E2* gene, which stops transcription of these genes and uncontrolled expression of *E6* and *E7* proteins that play a key role in cervical carcinogenesis [18].

### 2.1. Primary Immune Escape

The importance of immune system in cervical cancer may be proven on the example of patients with immune deficiencies. HIV infection compromises host immune response and accelerates cervical carcinogenesis. The risk of cancer in HIV-positive population is increased 10-fold (in comparison to seronegative women) [32]. HPV infections are twice as frequent in HIV-positive women, and SIL occurs in 36% of HIV-positive women [33]. Moreover, in 39% of HIV-positive women with NILM (negative for intraepithelial lesions or malignancy) Pap smear result, SIL is present in a cervical biopsy sample [34]. The first step in avoiding immune response is lack of the viremic phase in the early phase of HPV infection [35]. In the basal cell layer, the viral genome is amplified but maintained at low copy number (depending on the study between 50 and 200) [23, 36]. There is no cytolytic effect on the infected cells; thus, the viral DNA is maintained inside the cell nuclei (whether in an episomal form or integrated with the host genome form). This causes lack of cellular response in this early phase of infection [35], as well as impairs cytokine and interferon production by infected cells [37]. As an effect, Langerhans cells will not be recruited into the infected region, ultimately allowing persistent infection to flourish [38].

### 2.2. Lymphocyte Infiltration and E2 Oncoprotein Mediated Immune Shift

As progression to high-grade lesions occurs, CD8+ lymphocyte infiltration in the epithelium, as well in stroma, may be observed. The presence of CD8+ T cells is more frequent in regressing lesions supporting their positive role in self resolution of HPV infection [39]. Chemoattractants for myeloid cells and monocytes are present in the cells with higher risk of progression [10]. This leads to infiltration with monocytes expressing high amounts of matrix metalloproteinase 9 (MMP-9) via CCR2 receptor and Ca^2+^ intracellular pathway, with MMP-9 being present in up to 100% of patients with HSIL or SCC [40, 41]. Another pathway for MMP-9 expression is bromodomain-containing protein 4 (BRD4) phosphorylation dependent. BRD 4 is a bromodomain and extra terminal domain (BET) family member—a group of epigenetic proteins important for DNA-binding factors with an ability to modulate transcription [42]. This pathway is activated by E2 protein of high-risk (HPV16 and HPV 18) genotypes [43]. This effect is not observed in the presence of low-risk-type-derived E2 protein.

### 2.3. JAK/STAT and Interleukin Crosstalk

HPV-inflicted inflammatory response of the stroma may also induce cell proliferation through IL-2. Cervical cancer cells were proven positive for interleukin 2 receptor (IL-2R) subunits *α*, *β*, and *γ*, with an ability to induce the JAK/STAT signalling pathway, essential for cell division [44]. However, phosphorylation and activation of JAK/STAT proteins were observed for low levels of IL-2 and were opposite for high levels of IL-2 [45]. High levels of IL-2 induce cell cycle arrest (G1 phase) in cervical cancer cells, decrease cell proliferation but also reduce their sensitivity to cisplatin [46]. Along with ongoing persistent infection, HPV-infected cells produce CCL20 (mediated by high levels of interleukin-6 (IL-6)) a chemoattractant for T-helper-17 (Th17). Infiltration with Th17 lymphocytes has an allowing effect for chronic inflammation to be maintained within regions of HSIL and eventually their progression to cancer [40], thus pointing Th17 infiltration and IL-6 presence in biopsy samples as another potential biomarkers for intraepithelial lesion persistence and risk of progression. IL-6 also induces increased levels of IL-23 (mainly p19 subunit) production by fibroblasts which further promotes the inflammatory process supporting oncogenesis in cervical lesions. Suppression of cytotoxic T cell by macrophages expressing programmed death-ligand 1 (PD-L1) is associated with worse prognosis or survival for both squamous cell carcinoma and adenocarcinoma of the cervix [10, 47].

### 2.4. Expression of E6 and E7 Oncoproteins: p53 Proteolysis and Lifting the RB Protein Suppression

**Figure 1:**
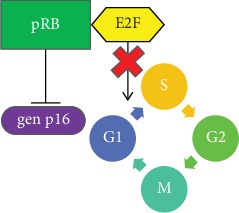
pRB role in cell cycle 1. During the G1 phase, unphosphorylated pRB forms a complex with E2F thus stopping progression to the S phase. Additionally, pRB has an inhibiting effect on p16INK4a gene promoter.

Retinoblastoma protein (pRB) is a suppressive protein, with an ability to arrest cell cycle [48]. In a normal cell, unphosphorylated pRB (active protein) forms a complex with E2F transcription factor and stops cell cycle progression from G1 to S phase (Figure 1) [49].

In HPV-infected cells where E6 and E7 proteins are expressed, this cell cycle control mechanism is lifted and cell replication is uncontrolled. The level of p53 rises after host cell DNA damage [50] but the ability to form E6-p53 complex (distinctive for HR HPV) [18] leads to ubiquitin-dependent p53 proteolysis and 2-3-fold decrease of p53 expression [51] slowing down DNA repair and proapoptotic processes, as well as increases cell proliferation. Another effect of E6 activity is telomerase activation allowing for indefinite number of cell divisions to take place, and together with lack of control mechanisms of cell proliferation, it occurs in an uncontrollable fashion [52].

**Figure 2:**
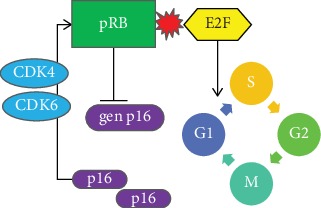
pRB role in cell cycle 2. P16 through its inhibiting effect on CDK 4 and 6 prevents phosphorylation of pRB. Hyperphosphorylated pRB cannot bind to E2F and allows it to promote progression to the S phase of the cell cycle.

E7 protein binds to unphosphorylated pRB and releases an active E2F transcription factor, initiating transcription and progression from the G1 phase to the S phase of the cell cycle [53] (Figure 2).

A suppressive pRB effect on p16INK4A is lifted, and overexpression of p16 protein may occur. Moreover, E7 inactivates p21 and p27 proteins and upregulates AKT activity [54]. AKT has a downstream product in c-myc which increased expression and activates telomerase activity adding effect to that of E6 [55]. Detection of p16 overexpression has an established position in cytological and histopathological diagnosis of cervical (and lower anogenital region) biopsy samples [18, 56], although there are pitfalls in abusing p16 immunohistochemical (IHC) staining, which may condone to a false-positive diagnosis [57].

### 2.5. JAK/STAT3 Expression in Cancer

After initial activation of JAK/STAT3 in high-grade intraepithelial lesions, development of cancer shifts those changes. Cancer cells lose receptor chain binding IL-6, and STAT3 is no longer expressed in cancer cell nests. This leads to induction of interferon regulatory factor 1 (IRF-1)—a proapoptotic transcription factor. With higher levels of IHC expression of IRF-1, response to chemotherapy and immunotherapy was observed to be better [58–60], thus pointing to IRF-1 as a potential biomarker for patient-tailored (personalized) therapy.

### 2.6. PI3K/AKT/mTOR Pathway Disruption by E6/E7 and Effects of Hypoxia

Phosphatidylinositol-3-kinase (PI3K)/protein kinase B (PKB)-AKT/mammalian target of rapamycin (mTOR) signalling pathway has a key role in cell cycle regulation and is crucial for surviving cellular stress. Its disruption plays an important role in tumor development [61]. With the presence of E6 and E7 oncoproteins, PI3K is activated and upregulates the EGFR and MAPK/ERK pathway [62]. The main correlation between the PI3K/AKT pathway activation and E7 presence is through inactivation of Rb and p27 [18, 60]. Ongoing carcinogenesis is dependent on constant presence of E6/E7, and lack of these oncoproteins may lead to cellular senescence [63]. This may be induced, for example, by ectopic expression of E2 protein, blocking E6 and E7 transcription, thus reactivating p53 and Rb, and through mammalian target of rapamycin complex 1 (mTORC1). Hypoxia-inducible factor 1-alpha (HIF1*α*) (overexpressed in cancer cells) is degraded by proteasomes. However, this was observed only for normoxic HPV-positive cells [64]. Hypoxia-induced effect of the PI3K/mTOR/AKT pathway is countered by increased activity of HIF1*α* which is also hypoxia dependent [65]. HIF1*α* through regulated in development and DNA damage response 1 (REDD1) inhibits mTORC1 signalling and stops senescence in case E6/E7 is repressed [65]. This proves that mTORC2 is still active in hypoxic environment, whereas mTORC1 shows susceptibility to oxygen shortage [66]. Inhibition of REDD1 may reactivate mTORC1 activity and induce cell entry into senescence [67]. Even though mTORC1 activator AKT is induced in hypoxic cells, it remains downregulated, which means that REDD1 inhibiting effect is more powerful than AKT-related activation of mTORC1 [65]. It is proposed that therapy targeting the PI3K/AKT/mTOR pathway will give better perspectives in treatment of HPV-related cancer [68]. The pitfall however in this approach may lie in the decreased cell metabolism induced by decreased mTOR activity, and E6/E7 repression allows evasion of senescence and instead enters dormant-like state which is reversible upon reoxygenation condoning to risk of recurrence. This also tampers sensitivity to chemotherapeutic agents and E6-/E7-targeting immunotherapy [67]; although mTOR-targeted therapy for HPV-related cancers is of interest by many scientist, we still need more data to support clinical use of this therapy [61, 69].

### 2.7. Aneuploidy and Proliferative Potential of Precancerous Cells

As the cell cycle becomes deregulated in precancerous lesions and cancer, it is widely agreed that aneuploidy will occur in those cells with a question whether it is an effect or the cause of carcinogenesis [70, 71]. Arguments for the aneuploidy to be an effect of cancerous progression are that chromosomal instability is often caused by p53 and pRB dysregulation, which is very important in HPV-related oncogenesis, and aneuploidy is the endpoint of the aforementioned chromosomal instability [72]. It was found that precancerous cells and CaSki cells have similar growth rate, higher than that of normal uterine cervix cells and higher percentage of aneuploid cells than normal cells. However, aneuploid and quadraploid cells constituted for about 25% of cells in normal cervical tissue vs. 35% and 37% in precancerous and CaSki cell lines, respectively [73]. The answer may lie in the ability of normal squamous epithelial cells to divide and constantly replace dead, superficial layer cells. This points out to the need for further inquiry in this problem, before a cutoff line may be drawn for the use of aneuploid cell rate in clinical management of precancerous lesions or prediction of cancer response to therapy.

## 3. Perspectives and Conclusions

Since the discovery of relation between HPV presence and carcinogenesis, we have learned a great deal of biology and molecular interplay between host cells and HPV genome. Introduction of prophylactic vaccines brought another breakthrough in decreasing HPV-related cancers. However, it will not be possible to completely eliminate HPV infections. This is why further understanding of HPV-related cellular physiology adjustments is a key point in developing new diagnostic and therapeutic methods. It is crucial to evaluate diagnostic methods to prevent overtreatment of patients with a high chance of self-resolution of HPV infection on the one hand and accurately planning treatment in patients with already developed cancer for achieving highest possible disease-free survival (DFS) on the other. It may prove to be difficult when taking into account that most HPV infections are caused by more than one genotype, which may alter molecular changes observed in vitro in artificial cell lines.

Detection of E6 and E7 mRNA presence is used in diagnosing HPV infection for some time now. Even though methods detecting HPV DNA are of the widest clinical usage, there is arising data pointing to detection of presence of p16 overexpression to be useful in increasing cytological primary screening accuracy.

Our conclusion is that the following markers are eligible for further inquiry in clinical conditions:

(1) Positive prognosis markers for precancerous lesions—high chance of regression—useful in young patients where surgical treatment could be postponed or dropped altogether
  - Presence of CD8+ T cells
(2) Negative prognosis markers for precancerous lesions—high chance of progression
  - MMP-9, Th17 lymphocyte presence, IL-6, and HIF1*α*
(3) Prognostic markers
  - PD-L1
(4) Predictive markers and targets for personalized therapy
  - IRF-1, IL-2, and PI3K/AKT/mTOR pathway

## Figures and Tables

**Table 1:** Natural history of abnormal Pap smear evolution.

CIN	Regression	Persistence of lesion	Progression
LSIL (CIN1)	60%	30%	10% (to HSIL)
HSIL (CIN2, CIN3)	30%	60%	10% (to invasive cancer)

Abbreviations: CIN: cervical intraepithelial lesions; LSIL: low-grade squamous intraepithelial lesions; HSIL: high-grade squamous intraepithelial lesions.

**Table 2:** Genotype of HPV and related effect.

HPV genotype	Effect
1, 2, 3, 4, 7, 10, 41	Benign skin lesions—mainly warts
6, 11	Low oncologic risk genotypes; LR HPV; condylomata acuminata—genital warts; slow progression of changes and recurrence of changes are observed
16, 18, 31, 33, 35, 39, 45, 51, 52, 56, 58, 59, 68, 73, 82	High oncologic risk genotypes; HR HPV; play a crucial role in pathogenesis of SIL and anogenital region cancers
5, 8, 14	Cause benign lesions in the type of *epidermodysplasia verruciformis*

Abbreviations: LR HPV: low-risk human papilloma virus; SIL: squamous intraepithelial lesions; HR HPV: high-risk human papilloma virus.

**Table 3:** HPV proteins and their role in viral life cycle.

Protein	Function
E1	Regulator of viral DNA replication. Only protein with enzymatic function. Downregulates host immune response genes.
E2	Initiates viral DNA transcription and partitioning of viral genome. Shifts host immune response.
E3	Ubiquitin ligase activity. Specific function uncertain.
E4	Expressed in middle and upper layers of the epithelium. Disruption of cell cycle and keratin organisation. Arrests cell growth allowing for viral amplification.
E5	Transmembrane protein with a transforming activity. HPV 16-derived E5 targets EGF receptor.
E6	Major oncoprotein. Binds to p53 and allows for its proteolysis.
E7	Major oncoprotein. Binds to RB allowing for E2F to promote cell cycle entry undisturbed.
E8	As E8^E2C limits viral transcription and DNA replication. Plays an important role in keeping a low copy number in undifferentiated squamous epithelium cells. At the same time is up- and downregulated by viral proteins.
L1	Forms the icosahedral capsid—main capsid protein. Has an ability to self-assemble spontaneously and form VLPs.
L2	Minor capsid protein. Plays a role in virus assembly, encapsidation of viral DNA and transport of the virus into the nuclei of infected cells.

Abbreviations: HPV: human papilloma virus; EGF: epithelial growth factor; VLP: virus-like particles.
